# Cellular Anti-Melanogenic Effects of a *Euryale ferox* Seed Extract Ethyl Acetate Fraction via the Lysosomal Degradation Machinery

**DOI:** 10.3390/ijms16059217

**Published:** 2015-04-23

**Authors:** Seung-Hwa Baek, In-Jeong Nam, Hyeong Seob Kwak, Ki-Chan Kim, Sang-Han Lee

**Affiliations:** 1 Department of Food Science & Biotechnology, Graduate School, Kyungpook National University, Daegu 702-701, Korea; E-Mails: micro340@hanmail.net (S.-H.B.); injounge90@gmail.com (I.-J.N.); kwakhs01@gmail.com (H.S.K.); kkc1380@hotmail.co.kr (K.-C.K.); 2 Department of Nano-Science & Technology, Graduate School, Kyungpook National University, Daegu 702-701, Korea; 3 Food & Bio-Industry Research Institute, Kyungpook National University, Daegu 702-701, Korea

**Keywords:** *Euryale ferox*, seed, melanin, tyrosinase, lysosomal degradation

## Abstract

The aim of this study was to investigate the effect of ethyl acetate fraction of *Euryale ferox* seed extracts (Efse-EA) on melanogenesis in immortalized mouse melanocyte cell line, melan-a. Efse-EA showed strong dose-dependent mushroom tyrosinase inhibitory activity. Treatment of melan-a cells with 30 μg/mL Efse-EA produced strong inhibition of cellular tyrosinase and melanin synthesis. Efse-EA significantly reduced the levels of melanogenesis-related proteins, such as tyrosinase, tyrosinase-related proteins 1 and 2, and microphthalmia-associated transcription factor. Because Efse-EA treatment reduced tyrosinase protein levels without changing its mRNA expression, we investigated whether this decrease was related to proteasomal or lysosomal degradation of tyrosinase. We found that chloroquine, a lysosomal proteolysis inhibitor, almost completely abolished both the down-regulation of tyrosinase and the inhibition of melanin synthesis induced by Efse-EA. These results suggested that Efse-EA may contribute to the inhibition of melanogenesis by altering lysosomal degradation of tyrosinase, and that this extract may provide a new cosmetic skin-whitening agent.

## 1. Introduction

*Euryale ferox* Salisb., a large aquatic plant, is the only species of the Euryale genera and *Nymphaeaceae* family that is native to India, Korea, Japan, Southeast Asia, and China [1]. In traditional oriental medicine, *E. ferox* seeds have been used to treat chronic diarrhea, leukorrhea, polydipsia, renal disease, ischemic heart disease, and mouth dryness [2,3,4]. Previous studies indicated that *E. ferox* seeds contained tannins, tocopherol polymers, fucosterol, resorcinol, pyrogallol, cyclic dipeptides, glucosylsterols, cerebrosides, and polyphenols [5,6,7,8]. *E. ferox* seed extracts have shown significant antioxidant activity, effects on cell-mediated immunity, neuroprotective effects, and cardioprotective properties, including the prevention of myocardial ischemic reperfusion injury [3,9,10]. The seed coat of *E. ferox* has also shown significant antioxidant and anti-fatigue activities *in vivo* [11]. Some research has investigated the antioxidant activity of this plant on the skin, when present in cosmetic preparations [8]. However, there are no previous reports of the effects of *E. ferox* seed extracts on the mechanisms involved in melanogenesis.

Melanin, the main pigment in mammalian skin, protects against the harmful effects of ultraviolet (UV) irradiation, oxidative stress, and DNA damage to the skin. Melanin is synthesized in melanosomes and subsequently transferred from melanocytes to the surrounding epidermal keratinocytes [12]. Melanogenesis is a complex process involving a series of enzymatically catalyzed chemical reactions and a variety of signal transduction pathways. Melanogenesis is initiated by the tyrosinase (EC: 1.14.18.1)-catalyzed oxidation of tyrosine to dopaquinone via the intermediate, 3,4-dihydroxyphenylalanine [13]. In the absence of thiols, the second enzyme in the pathway, tyrosinase-related protein 2 (TYRP-2; dopachrome tautomerase), enables the rapid conversion of dopaquinone to dopachrome, and then to 5,6-dihydroxyindole (DHI) or indole 5,6-quinone 2-carboxylic acid (DHICA). TYRP-1 (DHICA oxidase) then catalyzes the oxidation of DHICA [14,15]. Tyrosinase is a key enzyme in the melanogenic pathway, since it catalyzes the rate-limiting reaction. Tyrosinase is a binuclear copper enzyme that is ubiquitously distributed in plants and animals. This enzyme catalyzes the hydroxylation of phenols to catechols, as well as the oxidation of catechols to quinones. Copper is present at the active site of tyrosinase [16,17]. Therefore, tyrosinase inhibitors with copper chelating activity can regulate or inhibit melanin production in the skin and are used in cosmetic products developed to treat dermatological disorders associated with melanin hyperpigmentation [18,19]. UV radiation induces the formation of reactive oxygen species (ROS), in addition to stimulating melanogenesis and proliferation of melanocytes in the skin [20]. Therefore, ROS scavengers and antioxidants can produce skin-whitening effects. A recent study reported that aminoglycosides, which are redox-inactive compounds, reduced the melanin levels in human melanocytes and produced significant changes in the activities of cellular antioxidant enzymes [21].

In the present study, we partitioned *E. ferox* seed extract using ethyl acetate, and found that this fraction was a potent inhibitor of melanin biosynthesis in a melanocyte cell culture system. Furthermore, we also investigated the effects of this *E. ferox* seed ethyl acetate fraction (Efse-EA) on the mechanisms involved in melanin biosynthesis. To the best of our knowledge, this is the first study of this nature.

## 2. Results

### 2.1. In Vitro Antioxidant Activities of E. ferox Seed Ethyl Acetate Fraction (Efse-EA)

**Figure 1:**
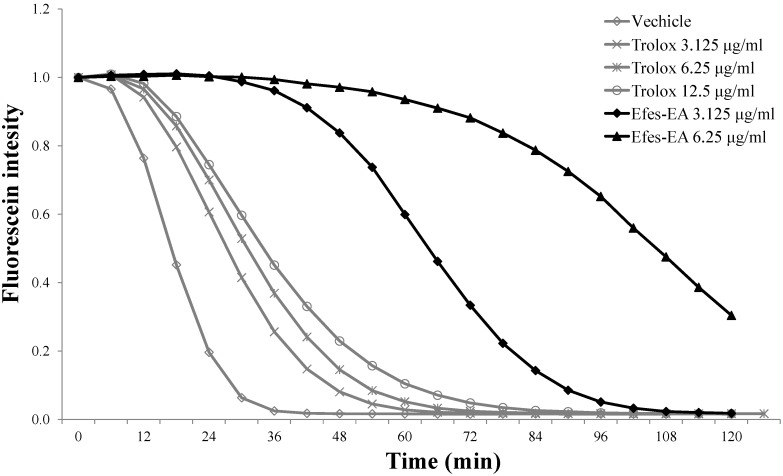
A kinetic curve of Efse-EA for antioxidant activity by oxygen radical absorbance capacity (ORAC) assay. The ORAC assay was carried out as described in Experimental Section. The final ORAC values were calculated using the regression equation for the Trolox concentration plotted against the net area under the fluorescence decay curve (AUC; area under the curve). Open triangle, vehicle; open diamond, Trolox 3.125 µg/mL; open circle, Trolox 6.25 µg/mL; closed circle, Trolox 12.5 µg/mL; closed diamond, Efse-EA 3.125 µg/mL closed triangle, Efse-EA 6.25 µg/mL.

The present study used several methods to determine the *in vitro* antioxidant potential of Efse-EA. The 1,1-diphenyl-2-picrylhydrazyl (DPPH) and 2,2'-azino-bis(3-ethylbenzothiazoline-6-sulfonic acid) diammonium salt (ABTS) radical scavenging assays have been used extensively to evaluate the free radical scavenging activities of antioxidants. Efse-EA and the positive control exhibited dose-dependent DPPH and ABTS radical scavenging activities (Table 1). The ferric-reducing antioxidant power (FRAP) assay measured the reduction of Fe^3+^ (ferric iron) to Fe^2+^ (ferrous iron) in the presence of antioxidants. This assay directly measured antioxidant effects on a redox-linked colorimetric reaction [22], in contrast to the other assays employed in the present study, which measured the inhibition of free radical generation in the reaction mixture [23]. Efse-EA caused a concentration-dependent increase in FRAP values from 20-fold to 820-fold (Table 1). The cupric(II) ion reducing antioxidant capacity (CUPRAC) assay was based on a single-electron transfer reaction, with the assumption that the antioxidant activity of the sample was equal to its reducing capacity. The Efse-EA had robust cupric reducing power, resulting in a significant 669-fold increase in the CUPRAC value at a concentration of 30 µg/mL (Table 1). The oxygen radical absorbance capacity (ORAC) assay measured the antioxidant-mediated inhibition of peroxyl radical-induced oxidation and thus reflected classical radical chain-breaking antioxidant activity by hydrogen atom transfer. The assay measures the loss of fluorescent intensity of molecules such as β-phycoerythrin or fluorescein over time in the presence of a constant flux of peroxyl radicals, generated from the thermal decomposition of AAPH in aqueous buffer. AAPH is the only free-radical generator that has been used in the ORAC assay to date [24]. The method uses an AUC (area under the curve) technique and thus integrates both the duration and degree of free radical inhibition by the antioxidant [23]. Figure 1 shows the kinetic curves for the Trolox positive control and Efse-EA. In our triplicate tests, the net AUC for Efse-EA was 86.1 at a concentration 6.25 µg/mL. Trolox had a net AUC of 6.3 at a concentration of 10 µg/mL. These results indicated that Efse-EA had greater antioxidant power than Trolox. Taken together, these results demonstrated that Efse-EA exhibited strong antioxidant activities using five different assay methods.

**Table 1:** Anti-oxidant activity of *E. ferox* seed ethyl acetate fraction (Efse-EA) in various *in vitro* assay systems.

Compound	Concentration (µg/mL)	DPPH ^(1)^	FRAP ^(2)^	CUPRAC ^(3)^	ABTS ^(4)^
		Activity (% of Control)			
Ascorbic acid	1	4.5 ± 0.011 ^(5)^	12.7 ± 0.002 **	12.2 ± 0.001	−1.7 ± 0.002
	3	8.5 ± 0.016 **^,(6)^	52.5 ± 0.001 **	42.7 ± 0.001 **	2.7 ± 0.001 **
	10	18.5 ± 0.054 *	170.8 ± 0.006 **	147.8 ± 0.001 **	18.1 ± 0.003 **
	30	57.3 ± 0.069 **	523.3 ± 0.026 **	119.2 ± 0.002 **	57.9 ± 0.008 **
Efse-EA	1	0.6 ± 0.002	19.9 ± 0.003 **	12.8 ± 0.003 *	5.2 ± 0.001 **
	3	7.9 ± 0.012	82.0 ± 0.009 **	54.3 ± 0.009 **	18.6 ± 0.013 *
	10	28.1 ± 0.006 **	234.4 ± 0.027 **	251.9 ± 0.029 **	55.1 ± 0.024 *
	30	78.0 ± 0.021 **	820.4 ± 0.065 **	669.6 ± 0.043 **	89.7 ± 0.001 *

^(1)^ 1,1-Diphenyl-2-picrylhydrazyl radical scavenging assay; ^(2)^ Ferric reducing antioxidant power assay; ^(3)^ Cupric(II) ion reducing antioxidant capacity assay; ^(4)^ 2,2'-azino-bis(3-ethylbenzothiazoline-6-sulfonic acid) diammonium salt radical scavenging activity assay; ^(5)^ Values are expressed as mean ± SD of triplicate determinations; ^(6)^ Data was considered to indicate statistical significance (* *p* < 0.05, ** *p* < 0.01) by means of the student *t*-test).

### 2.2. Effect of Efse-EA on Mushroom Tyrosinase and Copper Chelation

**Figure 2:**
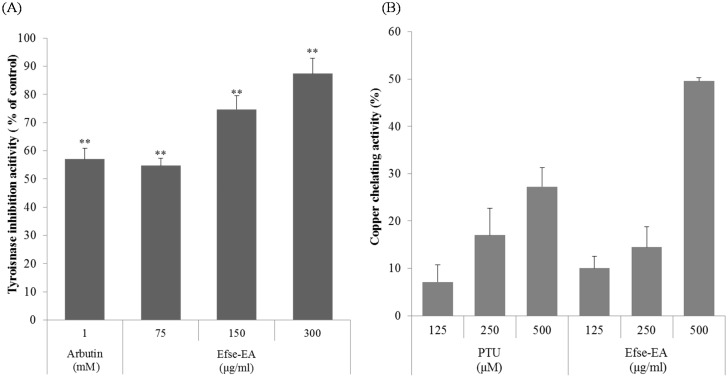
Anti-tyrosinase and copper chelating effects of Efse-EA. Tyrosinase was preincubated with the test substances at 25 °C for 5 min prior to incubation with l-tyrosine for 30 min; absorbance was read at 490 nm. Each determination was made in triplicate and the data shown represent the mean ± SD. ** *p* < 0.01, Student’s *t*-test (**A**); copper chelating effects were measured using pyrocatechol violet (PV) as described in Experimental Section (**B**).

We therefore examined the copper-chelating effect of Efse-EA, and its effects on tyrosinase activity, which is the key enzyme responsible for melanin synthesis. Figure 2A shows that Efse-EA inhibited mushroom tyrosinase activity in a dose-dependent manner. At a concentration of 300 µg/mL, Efse-EA inhibited mushroom tyrosinase by 87.4% ± 5.4% (Figure 2A, 4th column). Under the same conditions, the standard (arbutin) produced 57.0% ± 3.9% inhibition (Figure 2A, compare 1st and 4th columns). This indicated that Efse-EA was a powerful tyrosinase inhibitor, with effects comparable to those of arbutin. Moreover, Efse-EA exhibited 49.6% ± 0.7% copper chelating activity at a concentration of 500 µg/mL (Figure 2B, 6th column). The positive control, PTU, had a copper chelation rate of 27.2% ± 4.0% (Figure 2B, 1st to 3rd columns). These findings indicated that Efse-EA may inhibit tyrosinase by chelating copper.

### 2.3. Effect of Efse-EA on Cell Viability and Melanin Levels

**Figure 3:**
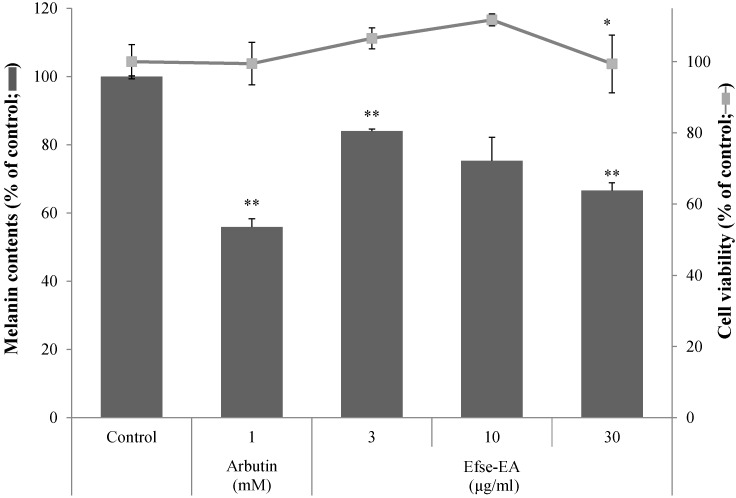
Effect of Efse-EA on melanogenesis in melan-a cells. Cells (1 × 10^5^ cells/mL) were seeded for overnight, and then the medium was replaced with fresh medium containing the indicated concentrations of Efse-EA or PTU (*N*-phenylthiourea). Cells were cultured for 72 h and further incubated for 24 h. After washing with PBS, the cells were lysed with 250 μL of 1 N NaOH and transferred to a 96 well plate. The melanin contents were estimated by measuring the absorbance at 405 nm. Each determination was made in triplicate and the data shown represent the mean ± SD. * *p* < 0.05, ** *p* < 0.01, Student’s *t*-test.

The level of melanin and cell viability of melan-a cells were examined following exposure to Efse-EA. Melan-a cells were incubated with Efse-EA (3–100 µg/mL) or arbutin (2 mM) for 5 days. At these concentrations, arbutin had no effect on cell survival (Figure 3, 2nd white column) but Efse-EA showed strong toxicity at 100 µg/mL (data not shown). Therefore, the melan-a cells were incubated with 30 µg/mL Efse-EA because this concentration was not associated with any cytotoxicity or morphological changes in the cells (Figure 3, solid line in graph). Following treatment with Efse-EA or arbutin, the melanin levels in melan-a cells decreased to 44.1% ± 2.5% and 33.4% ± 2.3% of that observed in the control group, respectively (Figure 3, 2nd to 5th black columns). These results indicated that Efse-EA had an inhibitory effect on melanogenesis in melan-a cells.

### 2.4. Effects of Efse-EA on l-DOPA (l-3,4-Dihydroxyphenylalanine) Zymography and on Cellular Tyrosinase Activity

**Figure 4:**
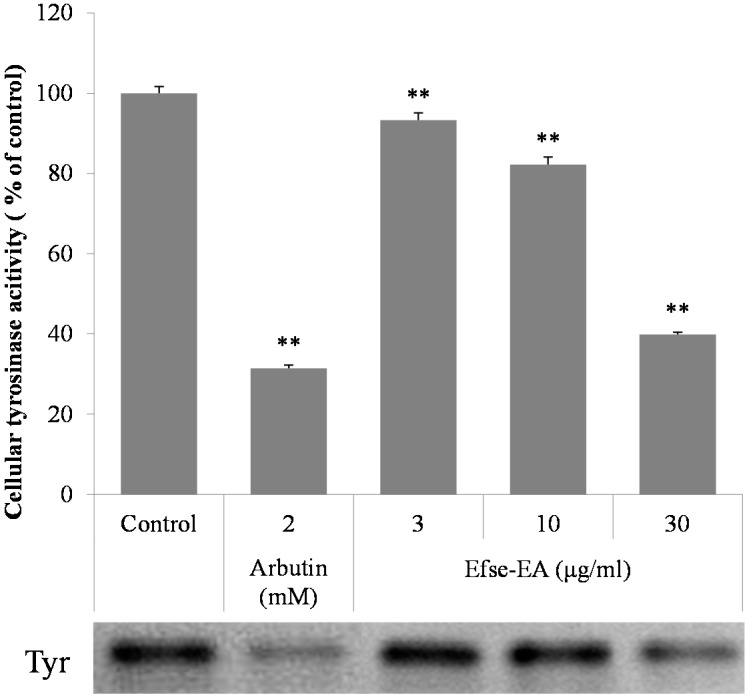
Effect of Efse-EA on intracellular tyrosinase activity. Melan-a cells (1 × 10^5^ cells/mL) were cultured for 24 h; the medium was replaced with fresh medium containing the indicated concentrations of Efse-EA or arbutin for 3 days. The cells were collected and lysed. After quantifying protein levels, tyrosinase activity was determined by l-DOPA zymography. Each determination was made in triplicate and the data shown represent the mean ± SD. ** *p* < 0.01, Student’s *t*-test.

The effects of Efse-EA on tyrosinase, measured by l-DOPA (l-3,4-dihydroxyphenylalanine) zymography, are shown in Figure 4. Treatment with Efse-EA at concentrations ranging from 3 to 30 µg/mL resulted in increasing inhibition of tyrosinase in melan-a cells (Figure 4, 3rd to 5th columns). Measurement of cellular tyrosinase activity showed that Efse-EA produced a potent anti-tyrosinase effect with an IC50 of 25.2 µg/mL; consistent with this, the concentration that inhibited l-DOPA zymography band density by 50% was about 27.7 µg/mL in melan-a cells. These results suggested that both l-DOPA zymography and measurement of intracellular tyrosinase activity identified the same effects of Efse-EA on intracellular tyrosinase activity.

### 2.5. Effect of Efse-EA on Proteins Involved in Melanogenesis and Lysosomal Degradation

**Figure 5:**
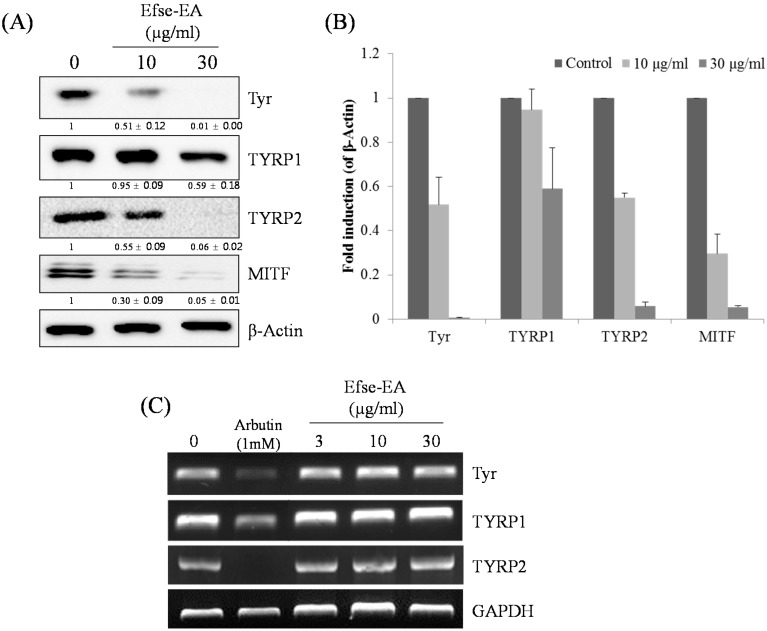
Effect of Efse-EA on the levels of melanogenesis-related mRNA and proteins in melan-a cells. Cells (1 × 10^5^ cells/mL) were cultured for 24 h; the medium was replaced with fresh medium containing the indicated concentrations of Efse-EA or arbutin for three days. Total cell lysates were extracted and assayed by Western blotting using antibodies against tyrosinase, tyrosinase-related protein (TYRP)-1, TYRP-2, and microphthalmia-associated transcription factor (MITF). Equal amounts of protein loading were confirmed using β-actin (**A**); Relative intensity of melanogenesis-related protein expressions, the intensity of the protein expressions was compared to the control; The normalized data for each were plotted as bar graphs (**B**); Cells (1 × 10^5^ cells/mL) were cultured for 24 h; the medium was replaced with fresh medium containing the indicated concentrations of Efse-EA or arbutin for 1 day. The mRNA was extracted using TRIzol; mRNA expression was analyzed by reverse-transcription polymerase chain reaction (**C**).

We examined the influence of Efse-EA on the levels of melanogenesis-related proteins by treating melan-a cells with Efse-EA for 72 h. We then examined the cell lysates using western blotting. As shown in Figure 5A, Efse-EA (30 µg/mL) reduced the levels of tyrosinase, TYRP-1, and TYRP-2 proteins by 100% ± 0.1%, 41% ± 0.2%, and 99% ± 0.2%, respectively, as compared with control cells (Figure 5A, 1st to 3rd rows. See Figure 5B for relative intensity). Additionally, Efse-EA significantly decreased microphthalmia-associated transcription factor (MITF) levels in these melanocytes (Figure 5A, 4th row). Melanogenesis has been reported to be associated with a down-regulation of ubiquitin-dependent proteasome degradation in melanocytes [25,26,27]. As Efse-EA treatment resulted in suppressed tyrosinase protein levels without changing mRNA expression (Figure 5C), it was likely that tyrosinase protein levels were being regulated by protein degradation.

**Figure 6:**
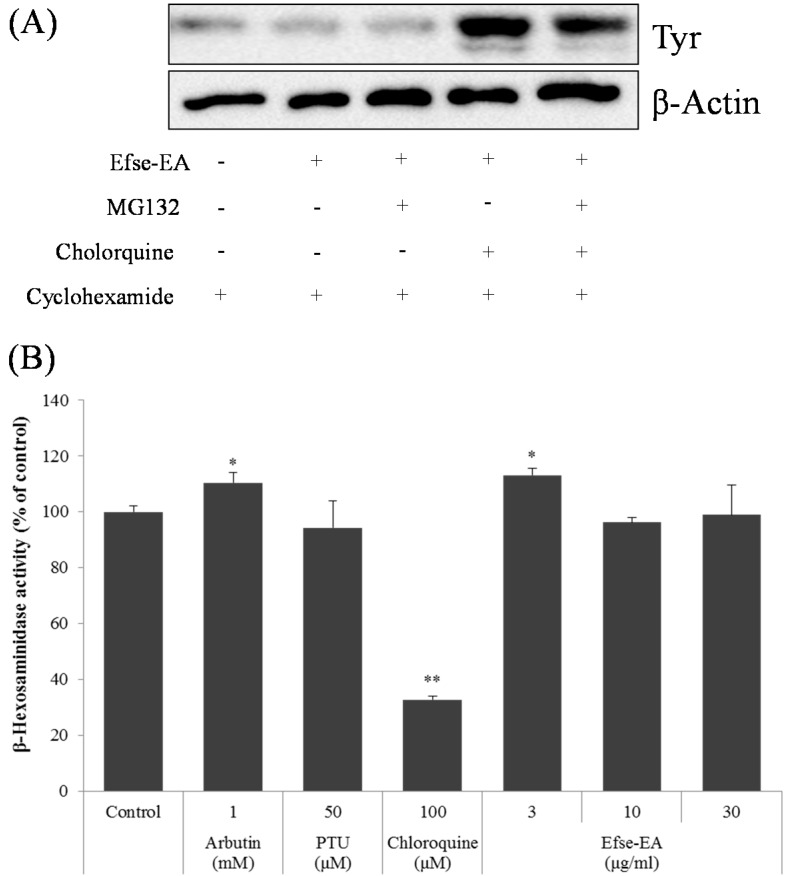
Effect of Efse-EA on lysosomal tyrosinase degradation in melan-a cells. Cells (3 × 10^5^ cells/mL) were pretreated with 25 μg/mL cycloheximide for 1 h, as indicated. Cells were also pretreated with 10 μM MG132 or 50 μM chloroquine for 1 h, and then treated with Efse-EA for 6 h. Whole cell lysates were subjected to western blotting using an anti-tyrosinase antibody. Equal protein loading was confirmed using actin (**A**); β-Hexosaminidase assay was calculated as described in Experimental Section. In brief, melan-a cells (2 × 10^5^ cells/well) were seeded and sensitized with 1 μg/mL of dinitrophenyl (DNP)-immunoglobulin E (IgE) and stimulated with 20 μg DNP-bovine serum albumin (BSA). Following 1 h incubation, supernatant was transferred and the substrate for β-hexosaminidase (1 mM 4-nitrophenyl *N*-acetyl-β-d-glucosaminide (NAG; N9376, Sigma) was added. After adding stop solution, the sample was measured at 405 nm with a spectrophotometer (**B**). * *p* < 0.05, ** *p* < 0.01.

Thus, to investigate whether the decreased level of tyrosinase reflected proteasomal degradation, MG132 (a proteasome inhibitor) and/or chloroquine (a lysosomal proteolysis inhibitor) were used. Cycloheximide was added to melan-a cells to inhibit protein synthesis, and they were then pretreated with MG132 and/or chloroquine for 1 h, followed by 6 h of Efse-EA treatment. As shown in Figure 6A, the decreased tyrosinase levels that occurred in response to Efse-EA were clearly restored by pretreatment with chloroquine. Conversely, treatment with MG132 did not affect the tyrosinase levels. To further investigate whether β-hexosaminidase, a lysosomal enzyme, is involved in lysosomal degradation, resulting in showing decreased enzyme level, we measured the enzyme activity by Efse-EA treatment in melanoma cells. Reluctantly, we could not find the inhibitory activity of Efse-EA, while chloroquine (100 µM) dramatically decreased the enzyme level (Figure 6B).

Taken together, these findings demonstrated that tyrosinase levels decreased due to lysosomal degradation, rather than proteasomal degradation, although the precise mechanism of lysosomal degradation is still undiscovered. As Efse-EA treatment reduced tyrosinase protein levels without changing its mRNA expression, it was likely that tyrosinase levels were regulated by protein degradation. These results clearly showed that Efse-EA may contribute to the inhibition of melanogenesis by regulating the expression of melanogenesis-related proteins and MITF.

## 3. Discussion

Traditional oriental medicine books refer to several skin-whitening herbs, most of which contain “*Baek*” as part of their Korean name. It has already been documented that *Mori cortex* Radicis (*Ssang-Baek-Pee* in Korean), *Atractylodes japonica* Koidzumi (*Baek-Chul* in Korean), and *Beauveria bassiana* (*Baek-Gang-Jahm* in Korean) are currently used as cosmetic ingredients; Korean ginseng (*Baek-Sam*) has also been used for its whitening activity. Although the academic name of Efse-EA does not contain *Baek* in Korean, it has been used as a natural whitening agent since long ago.

Melanin is a protective biomaterial secreted from melanocytes. In human skin, complex regulatory processes control melanin pigmentation [13]. Following skin exposure to UV light, melanocytes produce melanin to protect the skin from damage arising from UV-induced DNA mutations, DNA repair errors, and cancerous tissue changes. During this process, tyrosinase plays pivotal roles in melanin production and the produced melanin is transferred to adjacent cells. However, in recent years, active compounds used as skin-whitening agents for treating or preventing hyperpigmentation disorders have caused the reduction in the natural non-stimulated pigmentation process, which led to hypopigmentaion in natural skin cells. This negative effect is due to their direct inhibition of tyrosinase [17]. Hyperpigmentation, as well as skin inflammation, can arise from hormonal abnormalities, genetic disorders, and UV irradiation [12]. These events can cause the skin to appear older by triggering the formation of skin spots or freckles, and ultimately cause skin inflammation. Therefore, in the course of screening for anti-melanogenic agents in foods or other natural materials, we identified Efse-EA as a potential tyrosinase inhibitor. To investigate this finding further, we performed antioxidant and anti-melanogenic experiments in melan-a cells. We found that Efse-EA not only exhibited strong anti-melanogenic activity against mushroom tyrosinase, but also affected the lysosomal degradation of tyrosinase.

Plant antioxidants reduce intracellular oxidative stress and protect against oxidative stress-related disorders/diseases. Several methods have been established to determine antioxidant activity and each method has its own advantages and disadvantages [28]. Before examining its anti-tyrosinase activity, we tested the antioxidant activities of Efse-EA using a range of assays (DPPH, FRAP, CUPRAC, and ABTS), and compared the findings with those generated using Trolox. Efse-EA exceeded the antioxidant potential of Trolox at the concentrations tested (Figure 1).

In the course of screening for whitening biomaterials, we found that *E. ferox* seed extract has strong potential in inhibiting melanin production as well as tyrosinase activity. Therefore, we investigated its cellular anti-melanogenic effects. As shown in Table 1 and Figure 1, we confirmed that the antioxidant activity of *E. ferox* seed extract was distinctly more potent than that of Trolox. We therefore hypothesized that its antioxidant activity could decrease inflammatory skin reactions by reducing tyrosinase activity and hence melanin production. Tyrosinase is a rate-limiting, multifunctional, glycosylated oxidase that contains two copper atoms in its active site [17]. We found that the tyrosinase-inhibiting and copper-chelating activity of *E. ferox* seed extract were correlated with the addition of Efse-EA in a dose-dependent manner, suggesting an antioxidant potential to induce whitening activity (Figure 2).

As mentioned earlier, melanin can be synthesized from L-tyrosine, L-DOPA, and dopaquinone by tyrosinases; racial differences determine the subsequent production of eumelanin or pheomelanin. In this process, tyrosinase plays a key role in melanin synthesis. Previous studies revealed that arbutin, phenylthiourea (PTU), and kojic acid showed potent inhibition of tyrosinase activity. Although kojic acid had the strongest activity, its use is limited because of its toxicity. Most tyrosinase inhibitors reduced melanin levels by suppressing the mRNA expression of melanin-related proteins such as tyrosinase, TYRP-1, TYRP-2, MITF, melanocortin 1 receptor (MC1R), MC3R, agouti signaling protein (ASIP), and mahogunin ring finger-1 (MGRN1) [29]. Furthermore, cyclic adenosine monophosphate (cAMP), stimulates melanogenesis mainly through activation of MITF, and leads to induction of tyrosinase relative protein expression. cAMP-induced melanogenesis has been reported to be mediated by a cAMP response element (CRE) promoter via the binding of CRE-binding protein (CREB) family transcription factors that are activated by protein kinase A [30]. MITF is also down-regulated by mitogen-activated kinsase (MAPK), phosphoinositide 3-kinase (PI3K)/Akt and glycogen synthase kinase 3β (GSK 3β). Especially, MAPK activation plays a leading part in phosphorylation of MITF at serine-73, which leads to ubiquitination and subsequent MITF degradation, finally decreasing tyrosinase-related proteins synthesis [25]. Our present results showed that Efse-EA treatment resulted in suppressed MITF protein. In addition, we confirmed that ethanol extracts of *E.* *ferox* seed treatment was not observed over-expression of tyrosinase, TYRP-1, -2 and MITF genes (data not shown). This suggests that the Efse-EA leads to the MITF degradation. Thus, we further examined the effect of Efse-EA on phosphorylation of MAPK, CREB and MITF during the progress of melanin biosynthesis.

*E. ferox* seeds are known as a herbal source for medicines used for their whitening activity in Korea and China, aiding the healthy appearance of the skin according to *BonChoTaekYoGangMok*, a Chinese medicinal encyclopedia. The present study showed that Efse-EA had significant antioxidant activity and anti-inflammatory effects. Previous findings suggested that *E. ferox* seeds had the potential to inhibit melanin synthesis, because nuclear magnetic resonance and mass spectral analysis indicated that its major constituents were fucosterol, resorcinol, pyrogallol, and 4-*O*-methylgallic acid [8]. Although compounds and/or extracts like those of and from *E. ferox* seeds have anti-melanogenic activities, no mechanistic studies have been conducted on how the specific signaling molecules work and what kind of anti-melanogenic mechanisms are involved in the affected tissues/cells.

Lysosomes are membrane-bound vesicles that contain numerous digestive enzymes, such as glycosidases, proteases, sulfatases, and other lysosomal proteins. The enzymes are excreted from lysosomes to destroy injured cells and defend the microenvironment of targeted cells. Therefore, it is assumed that the expression of lysosomal enzymes, such as β-hexosaminidase and β-mannosidase, is increased under conditions of excess melanogenesis, as shown in a previous study in which fibroblast 3T3 cells were transfected with the enzymes’ genes [31]. This finding is highly interesting because lysosomal enzymes play a key role in regulating the activity/expression of tyrosinase during the melanin production process. To prove this specific role for lysosomal enzymes in our system, we examined the potential involvement of a lysosomal degradation machinery targeting tyrosinase. First, we investigated whether lysosomal enzymes decreased tyrosinase expression and/or degraded tyrosinase directly by treating melan-a cells with Efse-EA during their melanin production. As shown in Figure 6B, we found that 30 µg/mL of Efse-EA did not inhibit β-hexosaminidase activity in these cells, while its enzymatic activity was strongly inhibited by chloroquine. Figure 6A shows that lysosomal degradation, not proteasomal degradation, was associated with recovered tyrosinase expression after treatment with chloroquine. These results strongly suggest that the melanin content was not affected by the inhibition of the lysosomal activity of β-hexosaminidase. Because we did not confirm the direct evidence for the involvement of the lysosomal enzymes in tyrosinase degradation, there are still potential mechanism(s) to be uncovered on tyrosinase’s lysosomal degradation. Further studies on cystinosin (a cystine/H^+^ symporter expressed in melanocytes) and the product of pink-eyed dilution locus (P protein in melanosomes) are needed to elucidate the molecular anti-melanogenic mechanisms underlying the control of eumelanin and/or pheomelanin.

## 4. Experimental Section

### 4.1. Materials

Arbutin, *N*-phenylthiourea (PTU), L-DOPA (L-3,4-dihydroxyphenylalanine), L-tyrosine, sodium hydroxide (NaOH), thiazolyl blue tetrazolium bromide (MTT), Tween-20, *O*-tetradecanoyl phorbol-13-acetate (TPA), 2,2'-azino-bis(3-ethylbenzthiazoline-6-sulfonic acid) (ABTS), mushroom tyrosinase, 2,2'-azobis(2-amidinopropane) dihydrochloride (AAPH), 6-hydrolxy-2,5,7,8-tetramethylchroman-2-carboxylic acid (Trolox), and 2,2-diphenyl-1-picrylhydrazyl (DPPH) were obtained from Sigma-Aldrich Co. (St. Louis, MO, USA). All other reagents and chemicals were high-grade and commercially available.

### 4.2. Cell Cultures

The melanocyte cell line, melan-a, was purchased from Dorothy C. Bennett (St George’s, University of London, London, UK). The melan-a cells were cultured in RPMI 1640 medium supplemented with 10% fetal bovine serum (FBS, Hyclone, Utah, UT, USA), streptomycin-penicillin (100 µg/mL each), and 200 nM TPA, a potent tumor promoter, at 37 °C in 5% CO_2_. Cells were passed every 3 days until a maximal passage number of 40. Confluent melanocyte monolayers were harvested using a mixture of 0.05% trypsin and 0.53 mM ethylenediaminetetraacetic acid (Gibco BRL, Grand Island, NY, USA).

### 4.3. Preparation of Plant Extracts

Dried and powdered *E. ferox* seeds (200 g) were extracted with 95% ethanol under reflux for 3 h (three times) and dried in a rotary vacuum evaporator. The residue (2.12 g) was suspended in 3 L of H_2_O, and then partitioned sequentially with *n*-hexane and ethyl acetate using separatory funnels in a stepwise manner. After vacuum filtration, the solutions were concentrated in a rotary vacuum evaporator and the *n*-hexane extract (590.1 mg) and Efse-EA (420.2 mg) were dissolved in methanol (MeOH) at a concentration of 100 mg/mL.

### 4.4. Cell Viability Assay

Cell viability was determined by MTT assay [32]. A range of Efse-EA concentrations were added to melan-a cells and incubated for 24 h. Next, 100 μL of MTT solution (5 mg/mL MTT in phosphate-buffered saline (PBS)) was added to each well, followed by incubation at 37 °C for 1 h. After removal of the MTT solution, 1 mL of dimethyl sulfoxide (DMSO) was added to the wells and mixed vigorously. Absorbance was determined using a microplate reader (VICTOR3, Perkin Elmer, Waltham, MA, USA) at 470 nm.

### 4.5. Evaluation of in Vitro Antioxidant Activities

The DPPH radical scavenging assay was conducted as described previously [33]. The antioxidant ferric reducing ability of Efse-EA, was estimated using a previously described assay, with a slight modification [34]. The cupric reducing antioxidant capacity of Efse-EA was determined using the cupric reducing antioxidant capacity (CUPRAC) method. The reduction of Cu(II) to Cu(I) was observed at neutral pH using copper(II)-neocuproine reagent as the chromogenic oxidant [35]. ABTS radical scavenging activity was evaluated using a published method, with minor modifications [36]. The method used for the ORAC assay was also adapted from a previously described assay, with minor modifications [37]. The final assay mixture (200 mL total volume) contained 400 mM fluorescein and 40 mM AAPH. Several dilutions of Trolox (0–25 mM in microplate wells) were used to generate the calibration curve. A freshly prepared AAPH solution was used for each experiment. The temperature of the incubator was set at 37 °C and the fluorescence was recorded every 2 min after the addition of AAPH. The final ORAC values were calculated using the regression equation for the Trolox concentration plotted against the net area under the fluorescence decay curve (AUC).

### 4.6. Measurement of Tyrosinase and Copper Chelating Activity

Tyrosinase activity was determined spectrophotometrically as previously described, with minor modification [38]. The reaction mixture used for the determination of the effects of Efse-EA on mushroom tyrosinase (EC 1.14.18.1) activity contained 150 μL of 0.1 M phosphate buffer (pH 6.5), 3 μL of Efse-EA sample, 36 μL of 1.5 mM L-tyrosine, and 7 μL of mushroom tyrosinase (2100 units/mL in 0.05 M phosphate buffer, pH 6.5) in a 96-well microplate (SPL, Pocheon, Korea). The mixture was measured at 490 nm to record an initial value. After incubation at 37 °C for 30 min, absorbance was measured again at 490 nm using a microplate reader (VICTOR3, Perkin Elmer). Tyrosinase inhibitory activity was then calculated using the following equation:

Inhibition activity (%) = [(A − B) − (C − D)]/(A − B) × 100
(1)

where A was the final absorbance of the control reaction (no Efse-EA present), B was the initial absorbance of the control reaction, C was the final absorbance of the reaction in the presence of Efse-EA, and D was the initial absorbance of this reaction.

Copper chelating activity was determined using pyrocatechol violet (PV) as described, with slight modifications [16]. The test sample of Efse-EA was mixed with 1 mL of 50 mM sodium acetate buffer (pH = 6.0) and 100 μL of 2 mM CuSO_4_. After 10 min of incubation at room temperature, 100 μL of 2 mM PV was added. After 20 min, the absorbance was measured at 632 nm (A632 nm). DMSO and PTU were used as the negative control and the standard metal chelator positive control for the assay, respectively. The copper chelating activity was calculated using the formula:

Copper chelating activity (%) = [1 − (A − B)/(C − D)] × 100
(2)

where A was the A632 nm of the Efse-EA sample with buffer, CuSO_4_, and PV; B was the A632 nm of Efse-EA sample with buffer and PV; C was the A632 nm of buffer with CuSO_4_ and PV; and D was the A632 nm of buffer and PV.

### 4.7. Melanogenesis Inhibition Assay in Melan-a Cells

Cells were seeded into a 24-well plate (BD Falcon, Bedford, MA, USA) at a density of 1 × 10^5^ cells per well and allowed to attach overnight. The medium was replaced with fresh medium containing a range of Efse-EA concentrations. Cells were cultured for 72 h and further incubated for 24 h. After washing with PBS, the cells were lysed with 250 μL of 1 N NaOH and transferred to a 96-well plate. The melanin level was determined by measuring the absorbance at 405 nm using a microplate reader (VICTOR3, Perkin Elmer). PTU was used as a positive control [39]. Inhibition of melanogenesis was then calculated using the following equation:

Melanogenesis inhibition (%) = (A − B)/A × 100
(3)

where A was the absorbance of cells treated with Efse-EA or PTU and B was the absorbance of control cells.

### 4.8. Analysis of Intracellular Tyrosinase by Zymography

Intracellular tyrosinase, separated by SDS-PAGE according to its molecular weight, can be detected as dark-colored dopaquinone following incubation with l-DOPA solution [14]. Tyrosinase zymography was performed as described previously [40]. The test sample of Efse-EA was added to melan-a cells (1 × 10^5^ cells/mL), the cultured cells were washed with PBS and harvested with RIPA cell lysis buffer, and supplemented with protease inhibitors. The amount of protein was determined using a BCA protein assay kit (Pierce Biotechnology, Rockford, IL, USA). An equal amount (40 µg protein) of each sample was mixed with zymogram sample buffer at 37 °C for 30 min. Samples were separated by 10% sodium dodecyl sulfate-polyacrylamide gel electrophoresis (SDS-PAGE). After electrophoresis, the gels were incubated in 0.1 M sodium phosphate buffer for 30 min with gentle shaking. The gels were then stained using 20 mM L-DOPA in 0.1 M sodium phosphate buffer at 37 °C for 1 h.

### 4.9. Reverse-Transcription Polymerase Chain Reaction (RT-PCR) Analysis

Total RNA was extracted from melan-a cells using TRIzol (Ambion, Austin, TX, USA), according to the manufacturer’s instructions [41]. The quality of the total RNA sample was evaluated by determining the ratio of the optical density at 260 nm to that at 280 nm. To prepare a cDNA pool from each RNA sample, total RNA (2 μg) was reverse transcribed at 42 °C for 90 min in the presence of oligo(dT) primers and reverse transcriptase (Roche Molecular Biochemicals, Mannheim, Germany). cDNA was amplified by PCR, where the reaction mixture was incubated at 94 °C for 5 min, followed by 19 cycles of 94 °C for 30 s, 56 °C for 30 s, and 72 °C for 1 min; followed by a 10 min elongation cycle at 72 °C using a PCR Thermal Cycler Dice TP600 (TAKARA Bio Inc., Otsu, Japan). The following oligonucleotide primers were used for mouse tyrosinase (forward, 5'-CCCAGAAGCCAATGCACCTA-3', reverse, 5'-ATAACAGCTCCCACCAGTGC-3'); mouse TYRP-1 (forward, 5'-GCTGCAGGAGCCTTCTTTCT-3', reverse, 5'-AAGACGCTGCACTGCTGGTC-3'); mouse TYRP-2 (forward, 5'-GGATGACCGTGAGCAATGGC-3', reverse, 5'-CGGTTGTGACCAATGGGTGC-3'); mouse MITF (forward, 5'-CAGGCTAGAGCGCATGGACT-3', reverse, 5'-CTCCGTTTCTTCTGCGCTCA-3'); mouse MC1R (forward, 5'-ATCCCAGATGGCCTCTTCCT-3', reverse, 5'-ACACCATGGAGCCACAGATG-3'); and mouse glyceraldehyde-3-phosphate Dehydrogenase (GAPDH) as an internal control (forward, 5'-GCGAGACCCCACTAACATCA-3', reverse, 5'-GAGTTGGGATAGGGCCTCTCTT-3'). The PCR products were separated on a 2% agarose gel in TBE buffer at 100 V for 40 min. After electrophoresis, the PCR products were visualized by ethidium bromide staining and the signal intensity of each band was quantified and normalized to that of GAPDH.

### 4.10. Western Blot Analysis

Melan-a cell lysates were prepared using a standard protocol, mixed with sample buffer (250 mM Tris-HCl (pH 6.8), 0.5 M DTT, 10% SDS, 0.5% bromophenol blue, 50% glycerol, 5% 2-mercaptoethanol), and denatured at 100 °C for 5 min. Sample proteins (20 µg) were separated by 10% SDS-PAGE. Following electrotransfer to nitrocellulose membranes (Whatman, Dassel, Germany), the membranes were incubated overnight with 5% skim milk and a range of antibodies. Anti-tyrosinase (C-19), anti-TYRP1 (G-17), anti-TYRP2 (D-18), anti-MITF, and β-actin antibodies (Santa Cruz Biotechnology, Inc., Santa Cruz, CA, USA) were used as primary antibodies. Anti-goat IgG-horse radish peroxidase (HRP) (Santa Cruz) and anti-mouse IgG-HRP (Santa Cruz) were used as secondary antibodies. The antigen-antibody reaction was detected using an ECL solution system (Perkin Elmer).

### 4.11. β-Hexosaminidase Assay

Melan-a cells (2 × 10^5^ cells/well) were seeded with minimum essential medium (MEM; 41500-034, Gibco, Carlsbad, CA, USA) in a 24-well plate (Corning Incorporated, Corning, NY, USA) and sensitized with 1 μg/mL of DNP-IgE (D8406, Sigma, St. Louis, MO, USA) at 37 °C, 5% CO_2_ overnight. Siraganian buffer—119 mM NaCl, 5 mM KCl, 0.4 mM MgCl_2_, 25 mM piperazine-*N*,*N*'-bis(2-ethanesulfonic acid), 5.6 mM d-glucose, 1 mM CaCl_2_, 0.1% bovine serum albumin (BSA), adjusted to pH 7.2 with NaOH—was prepared shortly before use. After washing wells twice with 500 μL of siraganian buffer, the cells were incubated in 180 μL of sample with buffer for 30 min. In order to stimulate the cells, 20 μg dinitrophenyl (DNP)-BSA (D5050, Biosearch Technologies, Inc., Novato, CA, USA) was added and incubated at 37 °C, 5% CO_2_ for 1 h. Following incubation, 50 μL of supernatant was transferred to a 96-well plate and the substrate for β-hexosaminidase (1 mM 4-nitrophenyl *N*-acetyl-β-d-glucosaminide (NAG; N9376, Sigma-Aldrich Co., St. Louis, MO, USA) in 0.1 M citrate buffer (pH 4.5) was added. After incubation at 37 °C for 1 h, 200 μL stop solution (0.1 M Na_2_CO_3_/NaHCO_3_) was added and the sample was measured at 405 nm with a spectrophotometer (VICTOR3; Perkin Elmer, Wellesley, MA, USA). β-Hexosaminidase release was calculated as the activity in the DNP-BSA treatment group compared to that in the control [42].

### 4.12. Statistical Analysis

Data were expressed as the mean ± standard deviation. Statistical significance was determined using Student’s *t*-test for independent means, in the Microsoft Excel program. The critical level for significance was set at *p* < 0.05.

## 5. Conclusions

We found that Efse-EA exerted anti-melanogenic effects in melan-a cells via a lysosomal degradation mechanism of tyrosinase. These findings and additional ongoing research will contribute to the development of natural whitening ingredients for cosmetic use; especially since the presently commercially available cosmetic ingredients are partially toxic to human skin while those from ethnobotanical sources have highly promising potential.

## References

[1] Song C.W., Wang S.M., Zhou L.L., Hou F.F., Wang K.J., Han Q.B., Li N., Cheng Y.X. (2011). Isolation and identification of compounds responsible for antioxidant capacity of *Euryale ferox* seeds. J. Agric. Food Chem..

[2] Kim Y.H., Lee M.J., Lee H.S., Kim J.G., Park W.H. (2011). Screening of antioxidative effect and suppressive effect of LDL oxidation of *Euryale ferox* Salisbury. Korean J. Orient. Physiol. Pathol..

[3] Das S., Der P., Raychaudhuri U., Maulik N., Das D.K. (2006). The effect of *Euryale ferox* (Makhana), an herb of aquatic origin, on myocardial ischemic reperfusion injury. Mol. Cell. Biochem..

[4] Han Z., Luo J., Kong L.Y. (2012). Two new tocopherol polymers from the seeds of *Euryale ferox*. J. Asian Nat. Prod. Res..

[5] Zhao H., Zhao S., Guillaume D., Sun C. (1994). New cerebrosides from *Euryale ferox*. J. Nat. Prod..

[6] Zhao H., Zhao S., Sun C., Guillaume D. (1989). Glucosylsterols in extracts of *Euryale ferox* identified by high resolution NMR and mass spectrometry. J. Lipid Res..

[7] Row L.C., Ho J.C., Chen C.M. (2007). Cerebrosides and tocopherol trimers from the seeds of *Euryale ferox*. J. Nat. Prod..

[8] Choo S.J., Kim Y.H., Ryoo I.J., Xu G.H., Yoo I.D. (2009). Application as a cosmeceutical ingredient of *Euryale ferox* seed extract. J. Soc. Cosmet. Sci. Korea.

[9] Puri A., Sahai R., Singh K.L., Saxena R.P., Tandon J.S., Saxena K.C. (2000). Immunostimulant activity of dry fruits and plant materials used in indian traditional medical system for mothers after child birth and invalids. J. Ethnopharmacol..

[10] Lee M.R., Kim J.H., Son E.S., Park H.R. (2009). Protective effect of extracts from *Euryale ferox* against glutamate-induced cytotoxicity in neuronal cells. Nat. Prod. Sci..

[11] Wu C., Chen R., Wang X.S., Shen B., Yue W., Wu Q. (2013). Antioxidant and anti-fatigue activities of phenolic extract from the seed coat of *Euryale ferox* Salisb. and identification of three phenolic compounds by LC–ESI-MS/MS. Molecules.

[12] Hideya A., Hirofumi K., Masamitsu I., Vincent J.H. (2007). Approaches to identify inhibitors of melanin biosynthesis via the quality control of tyrosinase. J. Investig. Dermatol..

[13] Hearing V.J. (2011). Determination of melanin synthetic pathways. J. Investig. Dermatol..

[14] Sato K., Takahashi H., Iraha R., Toriyama M. (2008). Down-regulation of tyrosinase expression by acetylsalicylic acid in murine B16 melanoma. Biol. Pharm. Bull..

[15] Chao H.C., Najjaa H., Villareal M.O., Ksouri R., Han J., Neffati M., Isoda H. (2013). *Arthrophytum scoparium* inhibitions melanogenesis through the down-regulation of tyrosinase and melanogenic gene expressions in B16 melanoma cells. Exp. Dermatol..

[16] Rao F., Yuting Z., Yiran G., Fang C. (2014). Antioxidant and tyrosinase inhibition activities of the ethanol-insoluble fraction of water extract of *Sapium sebiferum* (L.) Roxb. leaves. South. Afr. J. Bot..

[17] Chang T.S. (2009). An updated review of tyrosinase inhibitors. Int. J. Mol. Sci..

[18] Kim K.N., Yang H.M., Kang S.M., Kim D., Ahn G., Jeon Y.J. (2013). Octaphlorethol A isolated from *Ishige foliacea* inhibits α-MSH-stimulated induced melanogenesis via ERK pathway in B16F10 melanoma cells. Food Chem. Toxicol..

[19] Su T.R., Lin J.J., Tsai C.C., Huang T.K., Yang Z.Y., Wu M.O., Zheng Y.Q., Su C.C., Wu Y.J. (2013). Inhibition of melanogenesis by gallic acid: Possible involvement of the PI3K/Akt, MEK/ERK and Wnt/β-catenin signaling pathways in B16F10 cells. Int. J. Mol. Sci..

[20] Zi S.X., Ma H.J., Li Y., Liu W., Yang Q.Q., Zhao G., Lian S. (2009). Oligomeric proanthocyanidins from grape seeds effectively inhibit ultraviolet-induced melanogenesis of human melanocytes *in vitro*. Int. J. Mol. Med..

[21] Wrześniok D., Beberok A., Otręba M., Buszman E. (2013). Effect of streptomycin on melanogenesis and antioxidant status in melanocytes. Mol. Cell. Biochem..

[22] Halvorsen B.L., Holte K., Myhrstad M.C., Barikmo I., Hvattum E., Remberg S.F., Wold A.B., Haffner K., Baugerød H., Andersen L.F. (2002). A systematic screening of total antioxidants in dietary plants. J. Nutr..

[23] Payne A.C., Mazzer A., Clarkson G.J., Taylor G. (2013). Antioxidant assays—Consistent findings from FRAP and ORAC reveal a negative impact of organic cultivation on antioxidant potential in spinach but not watercress or rocket leaves. Food Sci. Nutr..

[24] Gülçin İ. (2012). Antioxidant activity of food constituents: An overview. Arch. Toxicol..

[25] Park S.H., Kim D.S., Lee H.K., Kwon S.B., Lee S., Ryoo I.J., Kim W.G., Yoo I.D., Park K.C. (2009). Long-term suppression of tyrosinase by terrein via tyrosinase degradation and its decreased expression. Exp. Dermatol..

[26] Goh M.J., Lee H.K., Cheng L., Kong D.Y., Yeon J.H., He Q.Q., Cho J.C., Na Y.J. (2013). Depigmentation effect of kadsuralignan F on melan-a murine melanocytes and human skin equivalents. Int. J. Mol. Sci..

[27] Bellei B., Maresca V., Flori E., Pitisci A., Larue L., Picardo M. (2010). p38 regulates pigmentation via proteasomal degradation of tyrosinase. J. Biol. Chem..

[28] Kim D.B., Shin G.H., Lee J.S., Lee O.H., Park I.J., Cho J.H. (2014). Antioxidant and nitrite scavenging activities of *Acanthopanax senticosus* extract fermented with different mushroom mycelia. Korean J. Food Sci. Technol..

[29] Hida T., Wakamatsu K., Sviderskaya E.V., Donkin A.J., Montoliu L., Lynn L.M., Yu B., Millhauser G.L., Ito S., Barsh G.S. (2009). Agouti protein, mahogunin, and attractin in pheomelanogenesis and melanoblast-like alteration of melanocytes: A cAMP-independent pathway. Pigment Cell Melanoma Res..

[30] Lee H.D., Lee W.H., Roh E., Seo C.S., Son J.K., Lee S.H., Hwang B.Y., Jung S.H., Han S.B., Kim Y. (2011). Manassantin A inhibits cAMP-induced melanin production by down-regulating the gene expressions of MITF and tyrosinase in melanocytes. Exp. Dermatol..

[31] Borovanský J., Mommaas A.M., Smit N.P., Eygendaal D., Winder A.J., Vermeer B.J., Pavel S. (1997). Melanogenesis in transfected fibroblasts induces lysosomal activation. Arch. Dermatol. Res..

[32] Hansen M.B., Nielsen S.E., Berg K. (1989). Re-examination and further development of a precise and rapid dye method for measuring cell growth/cell kill. J. Immunol. Methods.

[33] Yoshida T., Mori K., Hatano T., Okumura T., Uehara I., Komagoe K., Fujita Y., Okuda T. (1989). Studies on inhibition mechanism of autoxidation by tannins and flavonoids. V. Radical-scavenging effects of tannins and related polyphenols on 1,1-diphenyl-2-picrylhydrzyl radical. Chem. Pharm. Bull..

[34] Benzie I.F., Strain J.J. (1996). The ferric reducing ability of plasma (FRAP) as a measure of antioxidant power: The FRAP assay. Anal. Biochem..

[35] Apak R., Güçlü K., Ozyürek M., Karademir S.E. (2004). Novel total antioxidant capacity index for dietary polyphenols and vitamins C and E, using their cupric ion reducing capability in the presence of neocuproine: CUPRAC method. J. Agric. Food Chem..

[36] Adewusi E.A., Steenkamp V. (2011). In vitro screening for acetylcholinesterase inhibition and antioxidant activity of medicinal plants from southern Africa. Asian Pac. J. Trop. Med..

[37] Ou B., Hampsch-Woodill M., Prior R.L. (2001). Development and validation of an improved oxygen radical absorbance capacity assay using fluorescein as the fluorescent probe. J. Agric. Food Chem..

[38] Ishihara Y., Oka M., Tsunakawa M., Tomita K., Hatori M., Yamamoto H., Kamei H., Miyaki T., Konishi M., Oki T. (1991). Melanostatin, a new melanin synthesis inhibitor. Production, isolation, chemical properties, structure and biological activity. J. Antibiot..

[39] Bennett D.C., Cooper P.J., Hart I.R. (1987). A line of non-tumorigenic mouse melanocytes, syngeneic with the B16 melanoma and requiring a tumour promoter for growth. Int. J. Cancer.

[40] Elias P.M., Menon G., Wetzel B.K., Williams J.W. (2009). Evidence that stress to the epidermal barrier influenced the development of pigmentation in humans. Pigment Cell Melanoma Res..

[41] Rozen S., Skaletsky H. (2000). Primer3 on the WWW for general users and for biologist programmers. Methods Mol. Biol..

[42] Gahl W.A., Potterf B., Durham-Pierre D., Brilliant M.H., Hearing V.J. (1995). Melanosomal tyrosine transport in normal and pink-eyed dilution murine melanocytes. Pigment Cell Res..

